# Timing of Puberty and Late Pubertal Height in Saudi Schoolboys: Riyadh Puberty Study II

**DOI:** 10.1155/2022/4343596

**Published:** 2022-10-20

**Authors:** Ibrahim Al Alwan, Haifa Alfaraidi, Fahad Al Juraibah, Mohamed Al Dubayee, Amir Babiker, Waleed Tamimi, Dania Musalli, Manal Alsheikh, Motasim Badri

**Affiliations:** ^1^College of Medicine, King Saud bin Abdulaziz University for Health Sciences, Riyadh, Saudi Arabia; ^2^King Abdullah Specialized Children's Hospital, King Abdulaziz Medical city, Ministry of National Guard Health Affairs, Riyadh, Saudi Arabia; ^3^King Abdullah International Medical Research Centre, Riyadh, Saudi Arabia; ^4^College of Public Health and Health Informatics, King Saud Bin Abdulaziz University for Health Sciences, Riyadh, Saudi Arabia

## Abstract

**Objective:**

Puberty has a significant contribution to the final height. Therefore, it is crucial to understand the normal variations in the onset and tempo of puberty in a specific population. In this study, we aimed to provide normative data on the timing of puberty and late pubertal height (LPH) in Saudi schoolboys in Riyadh.

**Methods:**

This is a cross-sectional field study (2011–2013) including Saudi schoolboys (grades 1–12; aged 6 to 19 years). Schools were chosen to represent the population from urban and rural areas in the Riyadh region. Pubertal maturity staging for gonads was assessed by measuring testicular size using a Prader orchidometer and assessing the Tanner staging of pubic hair. The marginal mean age was calculated using regression analysis.

**Results:**

We recruited 1086 schoolboys. The estimated mean age of pubertal onset at G2 was 11.8 (95% CI 11.60–12.0) years, for gonadal development at G3 was 13.2 (95% CI 12.9–13.5), G4 = 15.0 (95% CI 14.7–15.2), and G5 = 16.1 (95% CI 15.9–16.3) years, and for pubic hair stage 2 (PH2) was 12.6 (95% CI 12.4–12.9) years. The estimated time from G2/PH2 to G5/PH5 was 4.3 and 3.9 years, respectively. At the onset of puberty, the mean height was 144.7 cm and it reached 167.8 cm at G5 with a pubertal height gain of 23.1 cm.

**Conclusion:**

Our data present the norms of the timing of puberty and LPH in Saudi schoolboys. Saudi adolescent males are shorter than some European and American comparatives mainly due to shortness during childhood. However, they could have shorter LPH than Turkish, Greek, Thai, and Japanese due to a less pubertal height gain.

## 1. Introduction

Puberty is a process that entails the development of secondary sexual characteristics and the attainment of reproductive maturity. It is not a distinct event but rather a series of changes that occur over a number of years at a particular rate known as the tempo of puberty [1–3]. Initiation of puberty is determined by both genetic and environmental factors [4–8], with the first physical sign of puberty in boys being an increase in testicular volume [9, 10] as measured with a Prader orchidometer [11]. Although the age of pubertal onset is mainly affected by the genetic background, it is also a reflection of the general health and nutritional status of the population [12]. Secular trends in the onset of puberty have been observed, particularly in girls [13–17]. However, the evidence regarding secular trends in puberty onset is not as strong in boys [9, 15, 18–20].

It is crucial to understand the normal variations in onset and tempo of puberty when caring for a specific patient population as the age of pubertal onset differs between ethnic groups. Studies from the United States have consistently shown that the age of pubertal onset is younger in African American children compared to white children [21–23]. This has clinical implications both from a diagnostic and therapeutic standpoint, as precocious puberty, especially in boys, necessitates further investigations to rule out an underlying pathological process such as intracranial tumors [24, 25]. Therefore, determining the normal age range for pubertal onset is essential to avoid pursuing unnecessary investigations and treatment. However, estimating the mean age of a given genital development is complicated by unobservable exact dates of entering and leaving a given stage of development [26].

Other implications for the age of onset of puberty include its impact on late pubertal height and consequently on the final adult height. There is a dual relationship between puberty and growth. Growth acceleration during the first few years of life may herald the early onset of puberty, with this being more apparent in girls [27, 28]. On the other hand, the event of puberty is a good reserve that contributes significantly to final adult height with a promoting growth effect of sex hormones on the growth plate [29, 30]. Nevertheless, the effect of age at pubertal onset on the final adult height is somewhat conflicting, as some studies suggested that the final adult height is inversely proportional to the age of puberty onset [30, 31], while others have found no association between age at puberty onset and final adult height [31, 32]. Interestingly, the effect of age at puberty onset may be different between boys and girls, with an earlier age at growth spurt onset in boys associated with greater final adult height and the opposite being true in girls [23, 33].

We assumed that Saudi adult males might be relatively short, compared to other populations, partially because of the effect of the timing of puberty on height gain during this period resulting in a shorter “late pubertal height” and ultimately affecting the final adult height [3, 33–36]. The aim of our study is to determine normative data for both onset and tempo of puberty in Saudi boys and to analyze the related changes in height during puberty.

## 2. Methods

This cross-sectional study was conducted between 2011 and 2013, as part of a puberty field study phase II, in schoolboys in Riyadh region, Saudi Arabia. The study included a population-representative sample of Saudi schoolboys from all areas of Riyadh (north, south, east, and west) region. A total of 1086 schoolboys from grades 1–12 aged 6 to 19 years were included in the study using a clustered sampling technique. Using information from the Ministry of Education and Ministry of Planning to establish a study sampling framework, schools were chosen to represent all grades of schooling (elementary, intermediate, and secondary), socioeconomic status, and geographical regions (rural and urban) in Riyadh. Informed consent was obtained, and parents completed information forms including their socioeconomic status, the child's date of birth, and health history. Students excluded from the study were those with a history of chronic illness and hormonal therapy or who refused medical examination.

All participants were examined by a pediatric endocrinology consultant or senior endocrine trainees. Data were collected using structured forms. Clinical information included growth parameters where height was measured using a wall-mounted stadiometer and reported to the nearest 0.1 cm. The pubertal maturity staging for the gonads was assessed using a Prader orchidometer, and testicular size was determined by palpation and manual isolation of the testes. Testicular volumes of 1–3 ml were classified as stage 1, 4–8 ml as stage 2, 9–12 ml as stage 3, 13–15 ml as stage 4, and greater than 15 ml as stage 5, with stage 2 for gonadal development marking the start timing of puberty. Pubertal maturity staging for pubic hair was performed using Tanner staging ranging from stage 1 (prepubertal) to stage 5 (complete pubertal development). A systemic examination was also performed to look at any signs that could indicate a chronic disorder.

### 2.1. Statistical Analysis

Continuous data were described as means (standard deviation) and categorical data as frequencies (%). Age tabulations were produced with the attainment of stage 2 to stage 5 for the gonads and pubic hair, demonstrating the age limits of normal puberty in our study population. The analysis was stratified further by age for BMI and geographical region. Using regression analysis, two random coefficient mixed-effect models were fitted to calculate the marginal mean age. The first was fitted to calculate mean age by the gonad stage and the second by pubic hair. In both models, age was included as a continuous dependent variable, geographical area (rural vs urban) as a random effect variable, and BMI-for-age as a continuous covariate. The data were analyzed using SPSS, version 21 (IBM, NY, US).

## 3. Results

The study included 1086 schoolboys.  Table 1 describes the number and percentage of boys by age and their distribution in each of the gonadal and pubic hair stages as well as per body mass index (BMI). The estimated mean age at pubertal onset was 11.8 (95% CI 11.6–12.0) years. The estimated mean age for gonadal development at G3 was 13.2 years (95% CI 12.9–13.5) and was 15.0 years (95% CI 14.7–15.2) and 16.1 years (95% CI 15.9–16.3) at G4 and G5, respectively (Table 2). The time from G2 (onset of puberty) to G5 was 4.3 years. The mean age for pubic hair stage 2 (PH2) followed the G2 by 0.8 years (range 10.5–15.0 years). The mean ages for PH3, PH4, and PH5 were 14.3 years (95% CI 14.0–14.5), 15.3 years (95% CI 15.1–15.6), and 16.5 years (95% CI 16.3–16.7), respectively (Table 2). The time from the onset of the development of pubic hair to PH5 was 3.9 years.

At the onset of puberty, the mean height was 144.7 cm.  Table 3 shows the height changes in relation to gonadal (G) development in our cohort. The peak height velocity occurred at pubertal stages G3 and G4 in the study subjects, with an average height gain of 23.2 cm during the progression from G2 to G5 (Table 3).  Table 4 shows that the Saudi's mean height at G2 of 144.7 cm compared to American adolescents' height at G2 of 149 cm (with a 4 cm difference) had further increased by 2 cm during puberty making a difference of 6 cm in the LPH between adolescents in the two populations. The average height gain in our schoolboys from the onset of puberty (G2) to the attainment of G5 was 23.2 cm (Table 4). Compared to other nations in commonly used growth charts, such as CDC, WHO, and Dutch charts, the mean late pubertal height of Saudi adolescent males is lower than the mean final height reported for these populations (Table 5).

## 4. Discussion

This field study in schoolboys in the Riyadh region showed normative data on the timing of puberty with regards to onset and tempo and its effect on final adult height in Saudi schoolboys in comparison to findings from similar studies in other populations and to our previously published report, by the same research group, in 2006 [34]. We found that schoolboys in Riyadh enter puberty similar to most of the other populations in the age of onset and also similar to some of them in height at the onset of puberty. Nevertheless, Saudi subjects appear shorter than them later as adults. We determined the normative ranges for puberty onset and tempo in Saudi schoolboys from urban and rural areas in Riyadh. The height measurements at different stages of genital development were also taken to explore the dual relationship of puberty and growth, with not only the effect of timing of the pubertal growth spurt (PGS). A late pubertal height at G5, which is near the final height, was compared with previous data on the final height from the Saudi growth chart and from other commonly used growth charts appreciating that Saudi subjects are possibly shorter due to the effect of a lower rate of height gaining during puberty. However, the initial height at G2 was also useful in the assessment of variations between the Saudis and other populations in the effect of genetic potentials and prepubertal growth on LPH.

The comparison between various studies looking at pubertal onset in different populations can be challenging since the parameter used to define pubertal onset may differ. This includes the use of testicular volumes of 4 ml or greater, attainment of G2 (which depends on testicular volumes, penile length, and scrotal skin texture), or onset of a pubertal growth spurt as an indicator of the onset of puberty [20, 37, 43]. In addition, different methods have been applied from direct inspection or inspection of a photograph, to determining testicular volumes using an orchidometer or by measuring their diameter, to self-reporting [9, 17, 24, 36, 38, 44]. In this study, we considered the attainment of G2 as an indicator of the onset of puberty. When comparing puberty-related studies, the mean age of pubertal onset in Saudi boys was similar to the age of pubertal onset in various Asian and Western populations. For example, the mean age of pubertal onset was 11.83 years in Danish boys in the early 1990s [19], 11 years in Greek boys in 1996 [45], and 11.3 years in boys from the Netherlands in 2001 [20]. Furthermore, the age of onset of puberty in Saudi boys is in keeping with what was described by Marshall and Tanner in 1970 with the mean age of onset of G2 being 11.6 years in boys from the United Kingdom [2]. The data described by Marshall and Tanner are frequently regarded as international references for pubertal staging. Our results were also in concordance with other populations in our region with the mean age of pubertal onset being 11.86 years in Egyptian boys [46, 47] and 11.6 years in Turkish boys [38]. Moreover, our results support using a classic age of 9 years as the cut-lower limit for normal puberty in Saudi boys. The estimated time from the start of puberty to its completion was approximately four years, which is similar to that in a Danish population study (4.1 years), longer than one of the US studies (3 years), and shorter than that reported in similar studies of other populations, namely, Turkish, Thai, Greek, and Japanese (4.9–6.2 years) [19, 39–41].

The second peak of growth in the most accepted infancy-childhood-puberty growth model occurs during puberty [48]. Pubertal height gain accounts for 17–18% of the final adult height [48]. The contribution to height gain during puberty is therefore much dependent on the onset time of puberty, the height at G2, the duration of puberty, and the potential of height gain during puberty [49]. Previous studies have reported that children with early puberty were taller during childhood and could be slightly shorter as adults compared to those who experienced normal or later maturation of puberty because they had limited time for their prepubertal growth [50–52]. This was the case when we compared our data on LPH with the data from adolescents in Greece, Turkey, Thailand, and Japan and also in the classic Tanner report in 1966, which is relatively old, with more or less similar or even shorter initial pubertal stature at G2 [38–42]. However, there were apparent variations in the initial height at G2 between the Saudis and adolescents from the Netherlands and the United States of America [9, 37]. Therefore, the differences in LPH between the Saudis and these latter groups from Europe and America were probably due to differences in prepubertal growth and the shorter stature of Saudis during childhood.

Pubertal height gain is variable in different populations depending on the pubertal maturity age (at G2 and G5 and the length of that duration) and the initial height at entering puberty (height at G2) besides the genetic potentials, nutrition, and other prepubertal factors that affect linear growth [9, 38–42, 44]. In our study, the mean height of schoolboys at the G5 pubertal stage was 167.8 cm. This is in the same range as the mean height of Saudi boys at pubertal stage G5 (169.36 cm), which was reported by our team in 2006 [34]. Both were in agreement with the reported mean height of a Saudi adult male of 168.8 cm by a national study [33]. The mean heights for male adults from the CDC and Dutch growth charts were 176.25 cm and 184 cm [3, 37], respectively. The shorter stature in Saudis could be explained by different possible factors, including genetic potentials and nutritional status; both affect growth before puberty as per the famous acceptable growth model, and also differences in height at the age of pubertal onset as well as the differences in pubertal height gain that could also be possibly genetically determined. In general, pubertal growth spurt (PGS) is used as a parameter for final adult height [53]. Although the late pubertal height (LPH) significantly contributes to final adult height (FAH), boys will continue to gain height until the age of 19.2 years, on average [54]. Unfortunately, the cross-sectional design of our study did not allow us to determine on the age of onset and the duration of PGS. We reported on schoolboy's LPH to the age of 17 years based on the differences in height measurements at G2 and G5, but not exactly on the FAH that should rely on an assessment of full PGS.

Since our previous study in 2006 was only 6 years earlier than this study, we will be unable to comment on secular trends and we have just compared our current results in the tempo of puberty to the Riyadh Puberty Study I in 2006 [34]. The mean age of boys in G2 was 11.4 years, compared with 11.8 years in our study, and the mean age of boys in G5 was 15 years compared with 16.1 years in our study. The mean ages of onset and completion of pubic hair development in the previous study were 11.4 years and 15.1 years, respectively, compared with 12.6 years and 16.5 years in our study. These findings suggest that there is a tendency towards a slightly later onset of puberty in Saudi boys; however, this was balanced by a longer duration of tempo to complete the puberty. The observed older age at the onset of puberty in our study may be related to the population sampled. The boys included in the first Riyadh puberty study were mainly students in urban areas of Riyadh; whereas our study included participants from both rural and urban areas. Differences between environmental factors, nutritional factors, and in socioeconomic status between urban and rural areas may have played a role. Saleh et al. observed that Egyptian boys living in urban areas with better socioeconomic status had a tendency towards the earlier onset of puberty [46]. Moreover, since obesity has been associated with the earlier onset of puberty, youth living in urban areas tend to have higher rates of obesity compared with their counterparts living in rural areas [55, 56]. Nevertheless, variations in the body mass index considering overweight/obesity in our sample were adjusted for in our statistical analysis. Comparing data from this study to our previous findings in 2006 is, however, limited by a relatively short duration of only 5 to 7 years between the two studies which does not allow for an accurate assessment of secular trends in puberty.

We adopted a careful assessment of testicular size measurement and pubic hair by a consistently trained team of data collectors; however, we did not use genital growth in our study. Of course, that was a limitation but it helped us to avoid interobserver variations in reporting on genital growth in a large cohort of schoolboys in a field study. While the lack of assessment of interrater reliability of testicular volume measurements could also be a limitation, the use of manual palpation adds to the strength of our study. In a cross-sectional design, the individual variation in the tempo of puberty and whether a participant is at the beginning, middle, or end of each stage of puberty were not possible to be assessed. However, we believe that given the large sample size and using mixed effect models, to adjust for regional and nutritional variations between study subjects, in calculating marginal mean age, we provide here normative data on the onset and progression of puberty in Saudi schoolboys in Riyadh.

## 5. Conclusion

We present normative data on the timing of puberty and LPH in Saudi schoolboys in Riyadh. Saudi adolescent males seemed to be shorter than some European and American adolescents not because of abnormal puberty but mainly because of relative shortness during childhood. The Saudis had less pubertal height gain; hence, a shorter late pubertal stature than adolescents in Turkey, Greece, Thailand, and Japan. Future larger and longitudinal studies that include prepubertal growth follow-up, and pubertal growth spurt could possibly allow better comparisons of final adult heights in these populations.

## 6. Consent

Written informed consent and assent were obtained from parents and patients, respectively.

## 7. Disclosure

Ibrahim Al Alwan and Haifa Alfaraidi are the first authors of this study.

## Figures and Tables

**Table 1:** Distribution of schoolboys in each age (year) per pubertal staging and per body mass index.

Age (years)	Number (%)	Distribution by number (%) of boys in each gonadal and pubic hair stage											Distribution by number (%) per body mass index	
		G1	G2	G3	G4	G5	PH1	PH2	PH3	PH4	PH5	Normal	Overweight	Obese
6	9 (0.8)	8 (3.1)	1 (0.40)	—	—	—	9 (2.1)	—	—	—	—	—	—	9 (1.3)
7	35 (3.2)	31 (11.9)	4 (1.6)	—	—	—	34 (7.8)	1 (0.6)	—	—	—	—	—	30 (4.3)
8	54 (5.0)	48 (18.5)	6 (2.4)	—	—	—	53 (12.1)	1 (0.6)	—	—	—	—	—	49 (7)
9	81 (7.5)	55 (21.2)	23 (9)	3 (2.3)	—	—	75 (17.1)	5 (3.1)	1 (0.8)	—	—	—	—	59 (8.4)
10	100 (9.2)	54(20.9)	39 (15.3)	6 (4.6)	1 (0.6)	—	86 (19.6)	13(7.9)	1 (0.8)	—	—	—	—	63 (8.94)
11	149 (13.7)	42 (16.2)	80 (31.4)	26 (19.7)	1 (0.6)	—	100 (22.8)	44(27)	5 (3.8)	—	—	—	—	100 (14.2)
12	132 (12.2)	14 (5.4)	65 (25.5)	27 (20.5)	21 (13.1)	5 (1.8)	50 (11.4)	48 (29.5)	25 (18.8)	9 (0.8)	9 (4.9)	—	—	92 (13.1)
13	120 (11.0)	6 (2.3)	19 (7.5)	39 (29.6)	31 (19.4)	25 (8.9)	21 (4.8)	32 (19.5)	32(24.1)	28 (2.6)	28 (15.3)	7 (0.6)	7 (4.1)	82 (11.6)
14	105 (9.7)	1 (0.4)	11 (4.3)	21 (15.9)	36 (22.5)	36 (12.9)	7 (1.6)	16(9.85)	33(24.8)	40 (3.7)	40 (21.9)	9 (0.8)	9 (5.3)	77 (10.9)
15	124 (11.4)	—	6 (2.4)	5 (3.8)	36 (22.5)	77 (27.5)	2 (0.5)	3(1.85)	21 (15.8)	45 (4.2)	45 (24.6)	53 (4.9)	53 (31.4)	53 (7.5)
16	95 (8.7)	—	1 (0.40)	5 (3.78)	22 (13.8)	67 (23.9)	1 (0.2)	—	12 (9)	37 (3.6)	37 (20.2)	45 (4.2)	45 (26.6)	47 (6.7)
17	52 (4.8)	—	—	—	9 (5.68)	43 (15.4)	—	—	3 (2.3)	17 (1.6)	17 (9.3)	32 (3.0)	32 (18.9)	25 (3.5)
18	19 (1.8)	—	—	—	2 (1.3)	17 (6.1)	—	—	—	6 (0.6)	6 (3.3)	13 (1.2)	13 (7.7)	11 (1.6)
19	11 (1.0)	—	—	—	1 (0.6)	10 (3.6)	—	—	—	1 (0.1)	1 (0.5)	10 (0.9)	10 (5.9)	8 (1.1)
Total (100%)	1086	259	255	132	160	280	438	163	133	183	169	705	187	194

BMI: body mass index, G: genital stage, and PH: pubic hair staging.

**Table 2:** Unadjusted and adjusted mean age by the puberty stage in our study.

Puberty stage	Unadjusted		Adjusted^*∗*^		5^th^ percentile	95^th^ percentile
	Mean	95% CI	Mean	95% CI		
G2	11.7	11.5–11.9	11.8	11.6–12.0	9.0	15.0
G3	13.0	12.8–13.3	13.2	12.9–13.5	10.0	15.4
G4	14.8	14.6–15.0	15.0	14.7–15.2	12.1	17.0
G5	16.0	15.8–16.2	16.1	15.9–16.3	13.0	19.0
PH2	12.6	12.4–12.8	12.6	12.4–12.9	10.5	15.0
PH3	14.2	13.9–14.4	14.3	14.0–14.5	12.0	17.0
PH4	15.3	15.1–15.5	15.3	15.1–15.6	13.0	18.0
PH5	16.5	16.3–16.7	16.5	16.3–16.7	15.0	19.0

^*∗*^Adjusted for body mass index for age and geographical area. G: genital stage and PH: pubic hair stage.

**Table 3:** Mean height at different stages of pubertal development in our study.

Pubertal Stage	Number of subjects	Mean height (cm)	SD
G2	255	144.7	9.3
G3	132	152.3	9.0
G4	160	162.0	8.0
G5	280	167.8	7.5

G: genital stage and SD: standard deviation.

**Table 4:** Mean age of pubertal maturation and mean pubertal height gain (in cm) in pubertal studies of different populations.

Variables of interest	Our study	Biro, et al. (US) [9]	Brix, et al. (Netherland) [37]	Bundak, et al. (Turkey) [38]	Jaruratanasi-rikul, et al. (Thailand) [39]	Pantsiotou, et al. (Greece) [40]	Yoshii and Tanaka (Japan)^*∗*^ [41]	Tanner, et al. (UK)^*∗∗*^ [42]
Age at G2 (Years)	11.7	12.18	11.1	11.6	10.6	10.3	12.38	11.5
Height at G2 (cm)	144.7	149.8	155	146.1	138 (2012) 139 (1994)^*∗∗∗*^	143.4	143.8	144.5
Age at G5 (Years)	16.0	15.19	15.2	16.5	16.2	17.1	17.63	16.0
Height at G5 (cm)	167.8	173.3	184	172.5	170.3 (2012) 167.0 (1994)	175.9	170.7	173.7
Duration of puberty (years)	4.3	3.01	4.1	4.9	5.6	6.8	5.25	4.5
Pubertal height gain	23.5	23.5	29	26.4	28.5–31.8	32.5	26.98	28.2

^*∗*^Average values were taken from 3 groups (GR1-3) in the study. ^*∗∗*^Tanner report, 1966, is of a standard study in history on growth and tempo of puberty. It is old and hence, secular trends should be considered in comparisons. ^*∗∗∗*^Data extracted from a figure in the article though was not exactly reported in these numbers in the article. G: genitalia stage.

**Table 5:** Late pubertal height (in cm) in our study compared to the final height in previous studies.

Height charts	Our study	Saudi chart 2 2005	CDC chart 2000	WHO chart 2007	Dutch chart 1997	Tanner study (1966)
3^rd^ centile	152.3	153.6	162	162.8	170.7	162.2
50^th^ centile	167.8	168.8	176.25	176.5	184	174.7
97^th^ centile	179.5	180.7	188.4	190.3	197.2	187.2

CDC: Centers for Disease Control and Prevention and WHO: World Health Organization.

## Data Availability

Data/publications of phase I Riyadh puberty study used to support the findings of this study are available from the corresponding author upon request.

## Abbreviations

CDC: Centers for Disease Control and Prevention
G: Gonadal development
NY: New York
PH: Pubic hair development
US: United States.
